# Multilayer stents affect the final diameter of aortic aneurysms and maintain renal artery patency for a short time in a swine experimental model

**DOI:** 10.6061/clinics/2021/e2812

**Published:** 2021-05-11

**Authors:** Anna Paula Weinhardt Baptista-Strazzi, Ricardo Aun, Igor Rafael Sincos, Allana M. Tobita, Maria Fernanda Cassino Portugal, Vitória Penido de Paula, Oskar Kaufmann, Nelson Wolosker

**Affiliations:** Hospital Israelita Albert Einstein, Sao Paulo, SP, BR

**Keywords:** Aortic Aneurysm, Thoracic, Stent, Endovascular, Experimental, Model

## Abstract

**OBJECTIVES:**

We sought to analyze the hemodynamic effects of the multilayer flow-modulated stent (MFMS) in Thoracoabdominal aortic aneurysms (TAAAs).

**METHODS:**

The hemodynamic effects of MFMS were analyzed in aortic thoracoabdominal aneurysms in experimental swine models. We randomly assigned 18 pigs to the stent or control groups and underwent the creation of an artificial bovine pericardium transrenal aneurysm. In the stent group, an MFMS (Cardiatis, Isnes, Belgium) was immediately implanted. After 4 weeks, we evaluated aneurysm sac thrombosis and renal branch patency by angiography, duplex scan, and morphological analysis.

**RESULTS:**

All the renal arteries remained patent after re-evaluation in both groups. Aneurysmal sac thrombosis was absent in the control group, whereas in the stent group it was present in 66.7% of aneurysmal sacs (*p*=0.061).

The mean final aneurysm sac diameter was significantly lower in the stent group (mean estimated reduction, 6.90 mm; *p*=0.021). The proximal neck diameter decreased significantly in the stent group (mean difference, 2.51 mm; *p*=0.022) and grew significantly in the control group (mean difference, 3.02 mm; *p*=0.007). The distal neck diameter increased significantly in the control group (mean difference, 3.24 mm; *p*=0.017). There were no significant findings regarding distal neck measurements in the stent group.

**CONCLUSION:**

The MFMSs remained patent and did not obstruct the renal arteries within 4 weeks. In the stent group, the device was also associated with a significant decrease in aneurysmal sac diameter and a large proportion (albeit non-significant) of aneurysmal sac thrombosis.

## INTRODUCTION

Thoracoabdominal aortic aneurysms (TAAAs) are difficult to diagnose and treat. The complexity of available treatment options incurs high mortality and morbidity rates (1), emphasizing the need to research less invasive strategies with minimal risk of sealing failure.

Flow-modulating stents, a more recent therapeutic concept, shift the focus of treatment from aneurysm exclusion to the reconstruction of the parent vessel. The aptly named multilayer flow modulator stent (MFMS, Cardiatis, Isnes, Belgium), unlike covered stents or endografts, allows blood flow through the stent mesh into intersected side branches, and modulates laminar flow within the aneurysmal sac, leading to thrombosis (2).

In the case of TAAAs, MFMSs simplify the surgical procedure, sparing the need for catheterization of several branches while maintaining their perfusion (3). The effectiveness of this principle has been previously demonstrated in *in vitro* (4,5) and *in vivo* (6,7) trials, and computational studies (8), and has been successfully demonstrated in a series of cases in both intracranial (9) and peripheral arteries (10-12), with growing experience in the aortic sites (13-17).

From clinical experience, MFMSs convey high rates of technical success in implantation and low mortality and morbidity (15,17-19). However, some authors have stressed the importance of indiscriminately applying the device (20). Moreover, visceral ischemia after readjustment of antiplatelet therapy and aneurysmal sac growth have been reported (21).

Although most reports show aneurysmal sac thrombosis, several of these studies have observed continued aortic enlargement in treated segments (15,17,22,23).

Considering this scenario, the present study sought to create an experimental swine model to analyze the hemodynamic effects of the MFM stent in TAAAs. Aneurysmal sac thrombosis, aortic diameter behavior, and patency of the visceral branches were assessed through angiography, intravascular ultrasound, and morphological analysis.

## METHODS

The study was conducted between November 2016 and April 2019 at the Center for Experimentation and Training in Surgery of the Albert Einstein Jewish Hospital in São Paulo, Brazil, which is accredited by the Association for Assessment and Accreditation of Laboratory Animal Care. The study was approved by the institutional animal care and use committee. A completed Animal Research: Reporting *in Vivo* Experiments guidelines checklist is included in the Appendix.

Eighteen 4- to 10-month-old Large White pigs weighing between 37 and 74 kg were randomly assigned to either the test or stent group (n=9) or the control or no-stent group (n=9).

All animals underwent laparotomy to create an artificial transrenal aneurysm. In the stent group, an MFMS (Cardiatis, Isnes, Belgium) was implanted in all subjects immediately after the creation of the aneurysm.

After four weeks, we performed a re-evaluation by angiography and ultrasound and a second laparotomy to assess the aneurysmal sac growth. The aorta was explanted after euthanasia.

The procedures are detailed in the following sections.

### Preoperative anesthetic procedures

All pigs were restricted from consuming solids and liquids for a 12-hour period. All animals were intramuscularly anesthetized with ketamine (10.0 mg/kg) and midazolam (0.25 mg/kg).

The marginal ear vein was catheterized with a 22-gauge BD Insyte catheter (BD Infusion Therapy Systems Inc, Sandy, Utah) for venous access. The invasive arterial pressure was measured by catheterization of the right carotid artery.

Anesthesia was induced with etomidate (1 mg/kg) and propofol (5 mg/kg). Size 7.0 Portex endotracheal tubes (Smiths Medical, Ashford, UK) were used, and inhalational anesthesia was achieved with 1.5% isoflurane with a ventilator set at a tidal volume of 10 mL/kg. Anesthesia was maintained with fentanyl (2.5 mg/kg).

Fluid replacement was performed with a maintenance crystalloid solution at 10 mL/kg/h. Crystalloid solution at 1 to 2 mL/kg/h was administered in bolus form whenever necessary to maintain a mean blood pressure of at least 70 mmHg. The protocol dictated that animals with nonresponsive hypotension be excluded from the study.

Antibiotic prophylaxis was administered to all animals with penicillin G benzathine (2.4 million units IM) and cephazolin (1 g IV).

### Intraoperative Technique

**Supplementary Figure 1:**
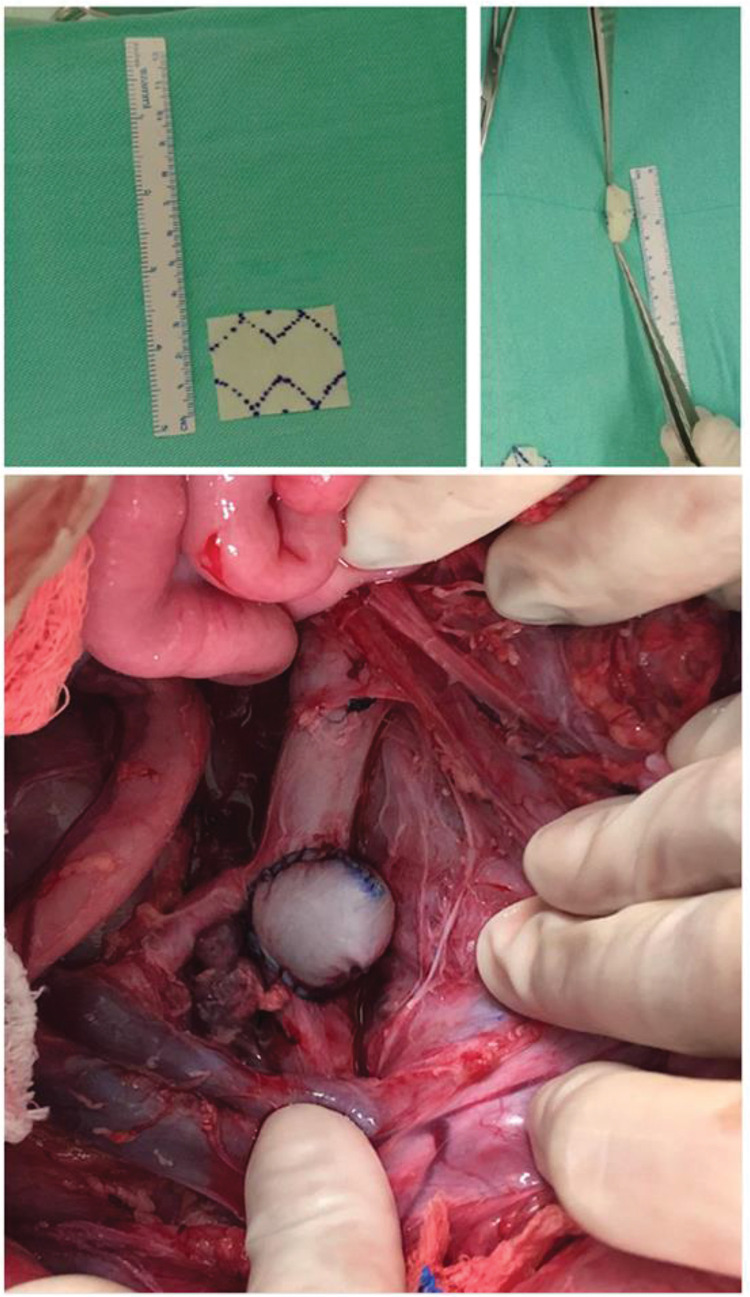
Back table aneurysm patch creation and aneurysmal sac final aspect.

In 16 cases, the aneurysmal patches were prepared on a back table prior to the start of the procedure: a bovine pericardial patch (Braile Biomedica, São José do Rio Preto, São Paulo) was folded in half and shaped into an oval configuration with a continuous suture pattern with Prolene 5.0, with approximate dimensions of 3 cm (length) × 2.5 cm (width) (Supplementary Figure 1).

In the two initial cases, the aneurysmal patch was constructed from the peritoneal tissue intraoperatively, following the same configuration and measurements. However, this technique prolonged the duration of the operation, leading to hemodynamic consequences and was therefore abandoned after the second case.

All animals were subjected to laparotomy with retroperitoneal dissection to access the visceral aorta with proximal (at the superior mesenteric artery level) and distal control (at the iliac bifurcation level). The renal and lumbar arteries were then repaired.

Systemic heparin (200 UI/kg) was administered to all subjects 2 min before proximal and distal clamping of the aorta.

The aorta was clamped proximally and distally to the renal arteries, and the renal and lumbar arteries were temporarily occluded. Once secure clamping was achieved, a longitudinal aortotomy of approximately 4.5 cm in length was done immediately distal to the superior mesenteric artery, with removal of an elliptic patch of the aortic wall measuring 3 mm. The aneurysmal patch, constructed from either the bovine pericardium or peritoneal tissue was then sutured to the aortotomy in a continuous suture pattern with Prolene 4 (Supplementary Figure 1).

After careful hemostatic revision, the aortic flow was restored, and the renal and lumbar arteries were unclamped.

Intraoperative aortography was performed immediately after flow restoration through the right femoral access via a 5F “*PigTail”* catheter (Impulse^TM^, Boston Scientific) with radiopaque centimeter markings positioned at the level of the first lumbar vertebrae. The aneurysm diameter and the extent and diameter of the healthy inframesenteric aorta were measured.

**Supplementary Figure 2:**
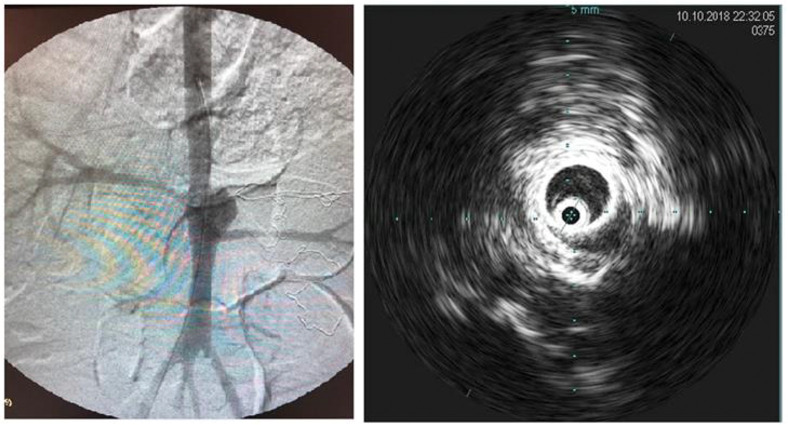
Aneurysmal sac and patent renal arteries in aortography (left) and IVUS assessment in a Stent group animal (right).

Immediately after aortography, imaging with intravascular ultrasound (IVUS) was performed for further comparison (Supplementary Figure 2).

Cases in which an enlargement of at least 50% of the healthy diameter of the aorta occurred in the aneurysmal section were considered technical successes.

### Stent group

In this group, an MFMS was selected in accordance with the aortic diameter as measured through IVUS and was implanted immediately after aortography through the right femoral access.

The stent was delivered just distal to the superior mesenteric artery, fully covering the aneurysm and renal arteries. Stent positioning and patency were verified using angiography.

### Postoperative protocol

Antiplatelet therapy was started once the animal could receive an oral diet (24-48h) with acetylsalicylic acid 100 mg orally once daily and maintained throughout the follow-up period.

### Re-evaluation reintervention

After 4 weeks, all surviving animals were subjected to a re-evaluation procedure consisting of IVUS and angiographic evaluation through femoral percutaneous access under general anesthesia, with additional conventional abdominal duplex scan evaluation when feasible.

Subsequently, all animals were subjected to laparotomy under the same protocol as the first surgery to assess new measurements of the aortic aneurysmal sac.

After aortic measurement, the animals were humanely euthanized under general anesthesia with a potassium chloride solution. The aorta was explanted in both groups.

### Stent evaluation and ultrasound analysis

All animals were evaluated by IVUS using a Volcano (Koninklijke Philips N.V., Amsterdam, The Netherlands) device and a digital catheter (Visions PV 0.35 or PV 0.18; Philips-Volcano).

When available, imaging was performed using a conventional transparietal duplex scan. However, this was limited because of residual pneumoperitoneum following laparotomy.

Imaging studies were performed to evaluate the final diameters of the aneurysmal sac and the aorta, assessment of thrombus formation in the aneurysmal sac, and verification of the patency of the renal arteries. In the stent group, they were additionally used to analyze stent positioning, device patency, and presence of endoleak.

### Aneurysmal sac and proximal and distal neck measurements

Multiple comparisons were performed separately between the control and stent groups for each procedure and between the “Aneurysm construction” and “Four-week re-evaluation” procedures for each group. These results are presented as the mean estimated differences, with *p*-values corrected using the Bonferroni method.

### Statistical Analysis

Categorical data were expressed as absolute frequencies and percentages, and continuous data were expressed as means with standard deviations and minimum-maximum values.

The frequency distribution was verified through histograms, boxplots, quartile comparison graphs, and the Shapiro-Wilk test.

Fisher’s exact test was used to investigate the association of categorical data between the study groups. Continuous data analysis was evaluated using the Student's t-test or the Mann-Whitney test as adequate.

Linear models were adjusted to assess the group and procedure effects on the proximal neck, aneurysmal sac, and distal neck measurements. This adjustment was in accordance with pre- and postoperative measurements in the same animal, considering the group and procedure effects as well as the interaction between group and procedure.

The results are presented as mean adjusted values with standard errors and 95% confidence intervals (CIs). The *p*-values obtained from multiple comparisons between measurements and group procedures were adjusted using the Bonferroni method.

All analyses were performed using SPSS Statistics for Windows (version 19.0; IBM Corp, Armonk, NY). Statistical significance was set at *p*<0.05.

## RESULTS

No significant population differences were observed between the two groups (Supplementary Table 1).

The operating time, clamping time, and the occurrence of anesthetic complications were also similar in both groups (one in each group: one nonresponsive hypotension and one hypoxemia due to glottic edema secondary to an expansive hematoma after carotid catheterization). Surgical, anesthetic, or postoperative complications did not differ between the groups. The results are presented in Table 1.

There were two immediate postoperative deaths. Among the animals included in the surveillance period, two died within 36h of the intervention due to reperfusion injury. Additionally, three animals with postoperative complications had to be euthanized before 4 weeks needed for the control re-evaluation procedure. Therefore, they were excluded from statistical analysis. The animal outcomes are summarized in Table 2.

### Renal artery patency

Renal arteries remained patent at the time of re-evaluation in 100% of the animals in both groups (Table 3).

### Aneurysmal sac thrombosis

**Supplementary Figure 3:**
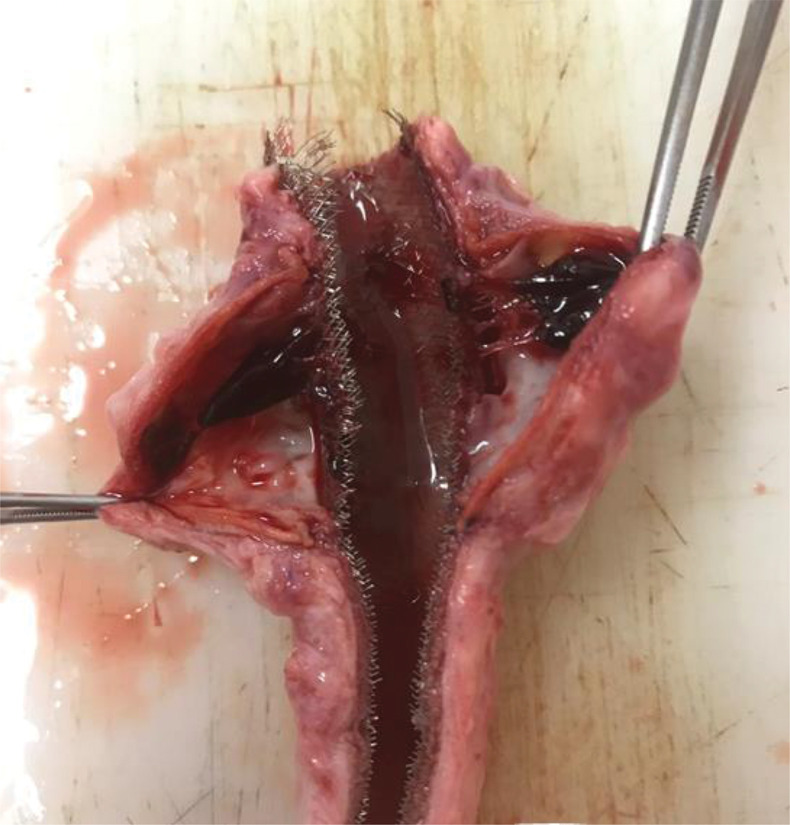
Aortic explant product with aneurysmal thrombus in the Stent Group.

The presence of a thrombus was primarily evaluated after aortic explanting through direct visualization of the vessel wall (Supplementary Figure 3).

In the control group (n=5), aneurysmal sac thrombosis did not occur on morphological evaluation and IVUS assessment (Table 1).

**Supplementary Figure 4:**
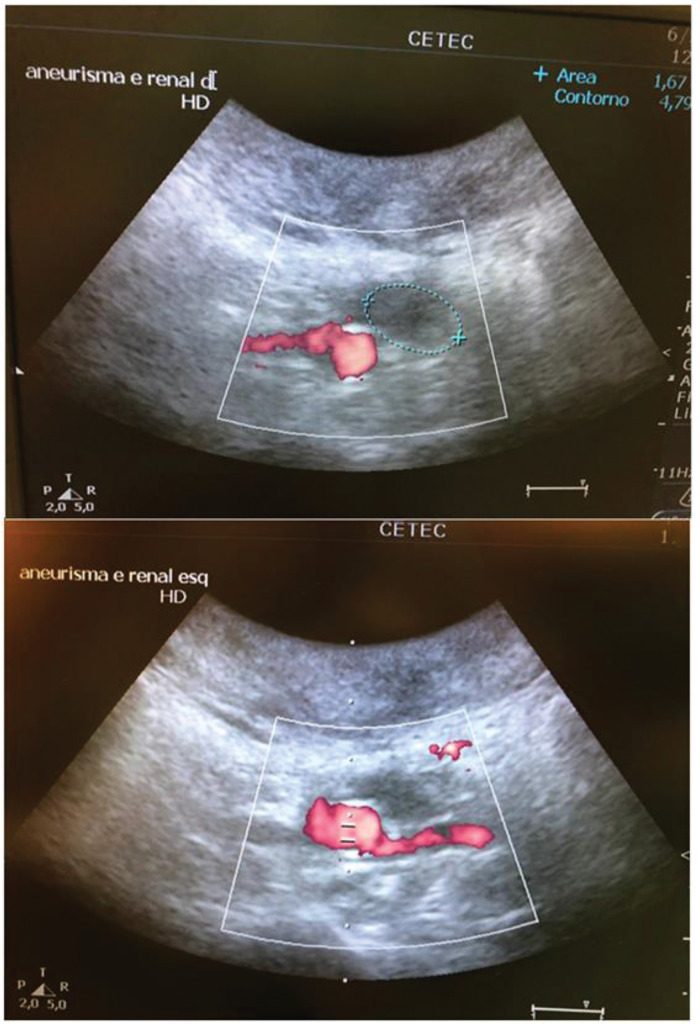
Aneurysmal sac thrombosis highlighted in blue, above. Patent right renal artery (above) and left renal artery (below) in a Stent Group animal, during duplex scan assessment.

In the stent group (n=6), aneurysmal sac thrombosis was seen after aortic explanting in 66.7% of cases; however, this was not confirmed by IVUS assessment (Table 1), possibly due to a technical limitation of the method regarding the echogenicity of the stent. However, when feasible, animals were subjected to a transparietal conventional duplex scan (Philips HD-11), and one animal in the stent group showed full aneurysmal sac thrombosis with patent renal arteries (Supplementary Figure 4).

Despite the notable number of sac thromboses in the stent group after aortic explanting, the difference between the groups was not significant (*p*=0.061).

### Aneurysmal sac, proximal and distal neck measurements

An association was observed between the study group and procedure to measure the proximal neck (*p*=0.058), aneurysmal sac (*p*=0.031), and distal neck (*p*=0.063), indicating that the procedure effect depends on the study group at the 10% significance level. Therefore, the test for multiple comparisons was conducted between the “Control” and “Stent” groups separately for each procedure and between the “Aneurysm construction” and “Four-week re-evaluation” procedures separately for each group. The results are presented in Table 3.

No intergroup differences were observed in the aneurysmal sac (*p*>0.999), proximal (*p*>0.999), or distal neck (*p*>0.999) measurements during the first procedure when the aneurysm was constructed.

The aneurysmal sac diameter in the stent group demonstrated a non-significant mean average decrease of 4.93 mm and a non-significant mean growth of 2.26 mm in the control group. When the mean differences between the groups at the final procedures were compared and adjusted by the Bonferroni method, a significant decrease was observed in the stent group, with a mean estimated reduction of 6.90 mm (*p*=0.021).

A significant decrease was also observed in the diameter of the proximal neck when comparing the stent group with the control group after 4 weeks (mean average difference, 2.51 mm; *p*=0.022). Regarding proximal neck measurements, there was also a significant growth in the control group from the first procedure to the re-evaluation procedure, with a mean difference of 3.02 mm (*p*=0.007).

The distal neck measurements in the control group demonstrated significant growth in 4 weeks, with a mean difference of 3.24 mm (*p*=0.017). There were no significant findings regarding the distal neck measurements in the stent group.

**Figure 1:**
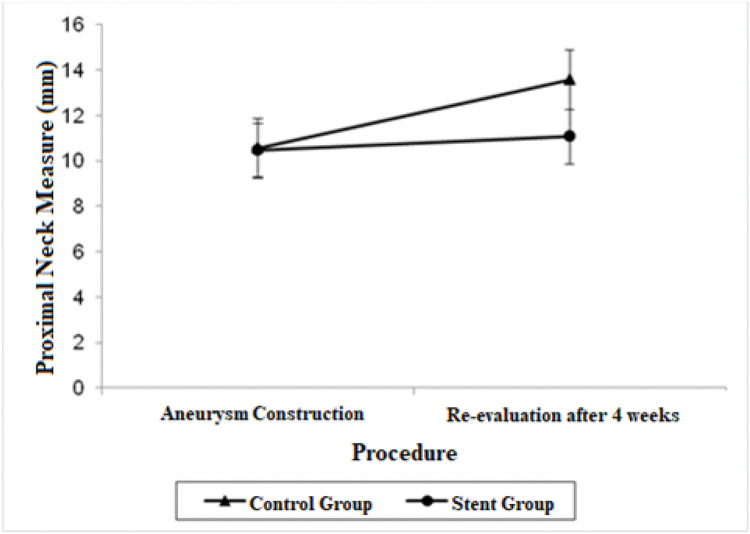
Mean adjusted values and 95% confidence intervals for proximal neck measurements (mm) in each procedure by group.

**Figure 2:**
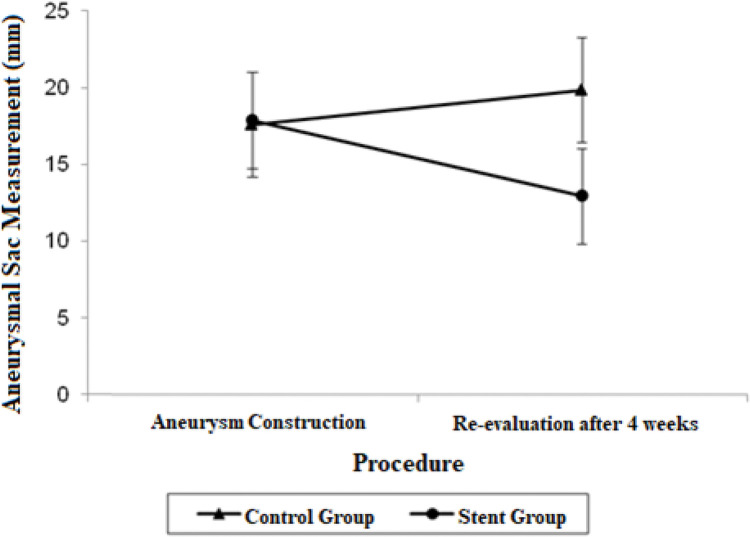
Mean adjusted values and 95% confidence intervals for aneurysmal sac measurements (mm) in each procedure by group.

**Figure 3:**
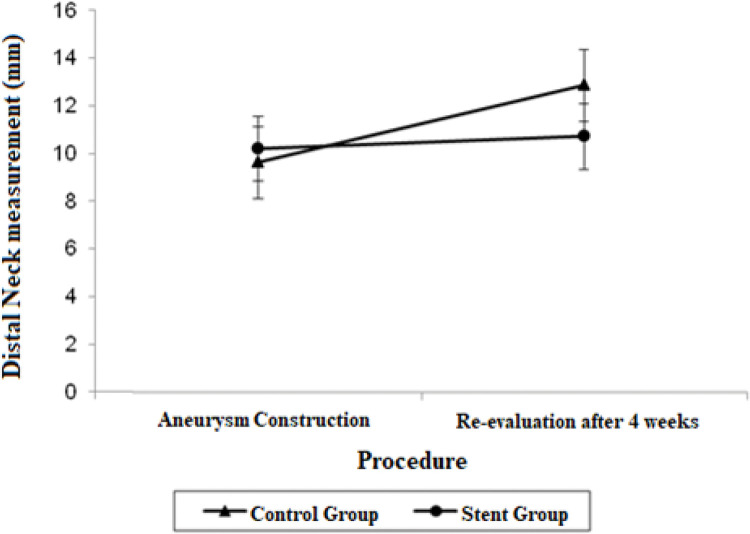
Mean adjusted values and 95% confidence intervals for distal neck measurements (mm) in each procedure by group.

The adjusted mean values and CIs for the measurements of the proximal and distal neck and aneurysmal sac in the two groups for each procedure are shown in Figures 1, 2, and 3.

Detailed results of the aortic explanting, angiographic and IVUS findings are described in Table 4.

## DISCUSSION

In this study, we developed an experimental porcine model to study the hemodynamic effects of MFMSs in artificially implanted saccular aneurysms in the aorta. Swine is used because it is an excellent biomedical model that closely replicates anatomical, physiological, and immunological conditions in humans (24-27).

Our sample demonstrated that the implantation of MFMSs did not lead to renal artery occlusion or stenosis, with patency rates of 100% at 4 weeks in both groups. Other authors have reported similar findings that side branch occlusions are rare in MFMS implantations and generally bear no clinical significance (13,15,17-19).

Thrombosis of the aneurysmal sac was primarily evaluated after aortic explanting. IVUS imaging and transparietal duplex scans were used as complementary methods, which both have limitations. Proper visualization of the aneurysmal wall was compromised with IVUS imaging because it was unable to transpose the echogenic metallic mesh of the stent. Conventional transparietal duplex scans are sometimes impaired due to the remaining pneumoperitoneum or bowel distension following laparotomy.

The incidence of sac thrombosis in the stent group was 66.7%. This difference was not statistically significant when compared to the proportion in the control group (*p*=0.061). The non-significance of this finding in our study may be due to our limited sample size or the relatively short follow-up interval. Follow-up was limited due to the proportionately restricted structure of our Center for Experimentation and Training in Surgery, in which keeping the animals longer could compromise their wellbeing due to their growth. In previous studies conducted by our group, we observed that at 4 weeks, animals had developed sufficiently for outcomes to be observed without jeopardizing animal handling due to their size or weight (24-26).

In a study by Oliveira et al. (6), manually manufactured multilayer stents composed of three metallic mesh stents joined together were implanted into swine models with aortic saccular aneurysms of bovine pericardium, in a similar fashion to our methodology, though not employing the Cardiatis MFMS. Although the authors did not evaluate aneurysmal sac thrombosis after aortic explants, a duplex scan was conducted on all subjects, and changes were noted in flow direction, from a whirlwind pattern in the aneurysmal sac to a laminar intra-stent flow, with remarkable differences in peak systolic speed (6).

In our study including 18 implanted aneurysms, 16 were constructed from bovine pericardium patches, and only two were constructed from the native peritoneum. The latter two were randomized to the control group. We believe this may be a cause for bias, considering that due to its allogeneic nature, the bovine pericardial patch was unlikely to present any growth.

In the stent group, however, we observed a significant decrease in the aneurysm size (*p*=0.021). This was accompanied by the aforementioned large proportion, albeit non-significant, of sac thrombosis.

Other findings regarding the aortic diameter in our study included a significant decrease in the proximal neck diameter in the stent group (*p*=0.022) as opposed to the significant growth of the proximal neck diameter in the control group (*p*=0.007), and a significant growth of the distal neck in the stent group (*p*=0.017).

These findings are unconventional among saccular aneurysms; however, aortic wall enlargement has been previously addressed in thoracoabdominal pathology reports, especially relating to dissections, suggesting that the reinstatement of laminar flow has a remodeling effect over the aortic walls (28). This could explain the findings of our study, but conclusive statements would require further investigation.

### Limitations

Due to the difficulties in establishing a proper intensive postoperative care system for swine models associated with the high mortality inherent in procedures of this type, we chose to restrict the sample size to the minimum necessary for statistical significance to limit animal mortality.

We incurred a possible bias by using a bovine pericardial aneurysmal patch since its allogeneic nature meant that it was unlikely to present any growth.

The mortality rate in this study (38%), which is consistent with that previously reported for the general treatment of TAAAs (29,30), was higher than that commonly observed in MFMS implantation (13,15,18,19,23,31). This deviation is likely due to the artificial implantation of aneurysms under supraceliacal clamping, which is inherently more invasive than customary MFMS implantation, and the practical and technical difficulties in establishing an adequate intensive intra- and postoperative care system for the swine models, leading to a relatively higher rate of postoperative and anesthetic complications.

## AUTHOR CONTRIBUTIONS

Baptista-Strazzi APW was responsible for study conception, data collection, manuscript composition, critical revision, manuscript acceptance, agreement to accountability. Aun R and Wolosker N were responsible for study conception, critical revision, manuscript acceptance, agreement to accountability. Sincos IR was responsible for study conception, data collection, critical revision, manuscript acceptance, agreement to accountability. Tobita AM and Kaufmann O were responsible for data collection, manuscript acceptance, agreement to accountability. Portugal MFC and Paula VP were responsible for manuscript composition, critical revision, manuscript acceptance, agreement to accountability.

## Figures and Tables

**Table 1:** Surgical characteristics of aneurysm creation procedure.

Procedure characteristics	Group		*p*-value
	Control (n=5)	Stent (n=6)	
Operation Time (minutes)			0.248^#^
Median (Q1; Q3)	195.0 (180.0; 210.0)	180.0 (180.0; 195.0)	
Min; Max	180; 220	130; 210	
Clamp time (minutes)			0.189^&^
Median (SD)	32.2 (11.7)	24.2 (6.9)	
Min; Max	18; 50	18; 35	
Aneurysmal sac thrombosis			0.061^§^
No (n [%])	5 (100.0)	2 (33.3)	
Yes (n [%])	0 (0.0)	4 (66.7)	
Surgical Complications			0.455§
No (n (%))	4 (80.0)	6 (100.0)	
Yes (n (%))	1 (20.0)	0 (0.0)	
Anesthetic Complications			>0.999^§^
No (n [%])	4 (80.0)	5 (83.3)	
Yes (n [%])	1 (20.0)	1 (16.7)	
Post-operatory complications			0.545^§^
No ( n [%])	3 (60.0)	5 (83.3)	
Yes ( n [%])	2 (40.0)	1 (16.7)	
IVUS visualization of aneurysmal sac thrombus			---
No ( n [%])	5 (100)	6 (100)	
Patency of renal arteries at re-evaluation procedure			---
Yes ( n [%])	5 (100)	6 (100)	

Q1: first quartile; Q3: third quartile; SD: standard deviation; ^#^: Mann-Whitney test; ^&^: t-Student test; ^§^Fisher's exact test.

**Table 2:** Individual animal outcomes.

Animal characteristics				
Animal study number	Group	Aneurysm Graft	Outcome	Survived until 4-week re-evaluation
Animal 01	control	peritoneum	Ischemia-Reperfusion injury, death before 4 weeks	no
Animal 02	control	peritoneum	Paraplegia followed by euthanasia, death before 4 weeks	no
Animal 03	control	bovine per.	Ischemia-Reperfusion injury, death before 4 weeks	no
**Animal 04**	**Stent**	**bovine per.**	**completed study protocol**	**yes**
Animal 05	control	bovine per.	Intestinal occlusion, death before 4 weeks	no
**Animal 06**	**control**	**bovine per.**	**completed study protocol**	**yes**
**Animal 07**	**control**	**bovine per.**	**completed study protocol**	**yes**
**Animal 08**	**control**	**bovine per.**	**completed study protocol**	**yes**
**Animal 09**	**Stent**	**bovine per.**	**completed study protocol**	**yes**
Animal 10	Stent	bovine per.	Expansive cervical hematoma, immediate postop. death	no
Animal 11	Stent	bovine per.	Nonresponsive hypotension, immediate postop. death	no
Animal 12	Stent	bovine per.	Paraplegia followed by euthanasia, death before 4 weeks	no
**Animal 13**	**Stent**	**bovine per.**	**completed study protocol**	**yes**
**Animal 14**	**control**	**bovine per.**	**completed study protocol**	**yes**
**Animal 15**	**Stent**	**bovine per.**	**completed study protocol**	**yes**
**Animal 16**	**control**	**bovine per.**	**completed study protocol**	**yes**
**Animal 17**	**Stent**	**bovine per.**	**completed study protocol**	**yes**
**Animal 18**	**Stent**	**bovine per.**	**completed study protocol**	**yes**

**Table 3:** Estimate mean values and 95% confidence intervals for aortic proximal neck, distal neck and aneurysmal sac at the aneurysm construction procedure and re-evaluation procedure.

Measurements (millimeters)	Control Group		Stent Group	
	Aneurysm Construction	Re-evaluation after four weeks	Aneurysm Construction	Re-evaluation after four weeks
	Estimate mean value (95%CI)	Estimate mean value (95%CI)	Estimate mean value (95%CI)	Estimate mean value (95%CI)
Proximal Neck	10.56 (9.25; 11.87)	13.58 (12.27; 14.88)	10.47 (9.27; 11.66)	11.07 (9.87; 12.26)
Aneurysmal sac	17.56 (14.14; 20.98)	19.82 (16.40; 23.24)	17.85 (14.73; 20.97)	12.92 (9.79; 16.04)
Distal Neck	9.62 (8.12; 11.12)	12.86 (11.36; 14.37)	10.20 (8.83; 11.57)	10.72 (9.35; 12.09)

95% CI: 95% Confidence interval.

**Table 4:** Angiographic and intravenous ultrasound assessment.

		Re-evaluation Procedure								
Animal Characteristics		Aortic Explant			IVUS			Angiogram		
Animal study number	Group	Patent Stent	Patent visceral branches	Sac thrombosis	Patent Stent	Patent visceral branches	Sac thrombosis	Patent Stent	Patent visceral branches	Sac thrombosis
Animal 01	control	_	yes*	no	_			_	yes* #	no* #
Animal 02	control	_	yes*	no	_			_	yes* #	no* #
Animal 03	control	_	yes*	no	_			_	yes* #	no* #
**Animal 04**	**stent**	**yes**	**yes**	**yes**	**yes**	**yes**	**not seen**	**yes**	**stent**	**yes**
Animal 05	control	_	yes*	no	_			_	yes* #	no* #
**Animal 06**	**control**	**_**	**yes**	**no**	**_**	**yes**	**no**	**_**	**yes**	**no**
**Animal 07**	**control**	**_**	**yes**	**no**	**_**	**yes**	**no**	**_**	**yes**	**no**
**Animal 08**	**control**	**_**	**yes**	**no**	**_**	**yes**	**no**	**_**	**yes**	**no**
**Animal 09**	**stent**	**yes**	**yes**	**yes**	**yes**	**yes**	**not seen**	**yes**	**yes**	**yes**
Animal 10	stent	_	_	_	_			yes* #	yes* #	no* #
Animal 11	stent	_	_	_	_			yes* #	yes* #	no* #
Animal 12	stent	yes*	yes*	no				yes* #	yes* #	no* #
**Animal 13**	**stent**	**yes**	**yes**	**no**	**yes**	**yes**	**not seen**	**yes**	**yes**	**no**
**Animal 14**	**control**	**_**	**yes**	**no**	**_**	**yes**	**no**	**_**	**yes**	**no**
**Animal 15**	**stent**	**yes**	**yes**	**yes**	**yes**	**yes**	**yes**	**yes**	**yes**	**yes**
**Animal 16**	**control**	**_**	**yes**	**no**	**_**	**yes**	**no**	**_**	**yes**	**no**
**Animal 17**	**stent**	**yes**	**yes**	**no**	**yes**	**yes**	**yes**	**yes**	**yes**	**no**
**Animal 18**	**stent**	**yes**	**yes**	**yes**	**yes**	**yes**	**yes**	**yes**	**yes**	**yes**

* Re-evaluation hastened by death or complication leading to euthanasia, these were excluded from statistical analysis.

# Aortic explant and/or angiograms were also performed in animals with hastened death to exclude occlusions in the aorta, side branches or the device in the stent group.

## Appendix

**Supplementary Table 1:** Population characteristics of animals subjected to aneurysm creation.

Animal characteristics	Group		p-value
	Control (n=5)	Stent (n=6)	
Age (months)			0.787^#^
Median (Q1; Q3)	4 (4; 4)	4 (4; 4)	
Min; Max	4; 10	4; 5	
Weight (kg)			0.413^&^
Median (SD)	53.8 (15.5)	47.5 (8.5)	
Min; Max	37; 74	40; 63	
Mean Arterial Blood Pressure (mmHg)			0.619^&^
Mean (SD)	82.4 (7.1)	86.8 (18.0)	
Min; Max	73; 90	64; 113	

Q1: first quartile; Q3: third quartile; SD: standard deviation; #: Mann-Whitney test; &: t-Student test
